# Bone Status in Patients with Epilepsy: Relationship to Markers of Bone Remodeling

**DOI:** 10.3389/fneur.2014.00142

**Published:** 2014-08-04

**Authors:** Sherifa A. Hamed, Ehab M. M. Moussa, Ahmad H. Youssef, Mohammed A. Abd ElHameed, Eman NasrEldin

**Affiliations:** ^1^Department of Neurology and Psychiatry, Assiut University Hospital, Assiut, Egypt; ^2^Department of Radiology, Assiut University Hospital, Assiut, Egypt; ^3^Department of Clinical Pathology, Assiut University Hospital, Assiut, Egypt

**Keywords:** antiepileptic drugs, bone mineral density, 25OHD, receptor activator of nuclear factor-kappa B ligand, osteoprotegerin

## Abstract

Patients with epilepsy and treated with antiepileptic drugs (AEDs) may develop metabolic bone disease; however, the exact pathogenesis of bone loss with AEDs is still unclear. Included were 75 adults with epilepsy (mean age: 31.90 ± 5.62 years; duration of treatment with AEDs: 10.57 ± 3.55 years) and 40 matched healthy controls. Bone mineral content (BMC) and bone mineral densities (BMD) of the femoral neck and lumbar spine were measured using dual-energy X-ray absorptiometry (DEXA). Blood samples were analyzed for calcium, magnesium, phosphate, alkaline phosphatase (ALP), 25-hydroxy vitamin D (25OHD), soluble receptor activator of nuclear factor-kappa B ligand (sRANKL), osteoprotegerin (OPG), and OPG/RANKL ratio (markers of bone remodeling). Compared to controls, patients had lower BMD, BMC, *Z*-score, and *T*-score at the femoral neck and lumbar spine (all *p* < 0.001). Seventy-two percent and 29.33% of patients had osteoporosis of the lumbar spine and femoral neck. Patients had significantly lower serum calcium, 25(OH)D, and OPG and higher ALP, sRANKL levels, and sRANKL/OPG (all *p* < 0.001). Fifty-two percent of patients had hypocalcemia, 93% had hypovitaminosis D, 31% had high levels of sRANKL, and 49% had low levels of OPG. No differences were identified between DEXA and laboratory results in relation to the type, dose, or serum levels of AEDs. BMD at the femoral neck and lumbar spine were found to be correlated with the duration of illness (*p* = 0.043; *p* = 0.010), duration of treatment with AEDs (*p* < 0.001; *p* = 0.012), and serum levels of 25(OH)D (*p* = 0.042; *p* = 0.010), sRANKLs (*p* = 0.005; *p* = 0.01), and OPG (*p* = 0.006; *p* = 0.01). In linear regression analysis and after adjusting for gender, age, weight, duration, and number of AEDs, we observed an association between BMD, 25(OH)D (*p* = 0.04) and sRANKL (*p* = 0.03) concentrations. We conclude that AEDs may compromise bone health through disturbance of mineral metabolism and acceleration of bone turnover mechanisms.

## Introduction

Epilepsy is one of the most common neurological disorders (1) and its treatment is life-long in nearly one-third of the patients (2). Several large epidemiological studies indicated that patients with epilepsy are at two- to six-folds increase in the risk of fractures compared to the general population (3). It has been reported that more than 15% of fractures are pathological due to metabolic bone disease (3, 4). Early reports suggested that institutionalization, inadequate dietary intake with vitamin D deficiency/insufficiency, reduced sunlight exposure (5), physical inactivity (6), aging (7), and female sex (8) are risk factors for osteopathy with epilepsy. However, many studies reported that antiepileptic drugs (AEDs) are major risk factors for bone disease in non-institutionalized patients and well-nourished ambulatory adults with epilepsy (9–12). Early reports incriminated AEDs with enzyme induction activity or their metabolism through hepatic cytochrome P450 enzyme system, as causes of metabolic bone disease due to increasing metabolism of vitamin D with secondary hypocalcemia and hyperparathyroidism (13–15). However, several studies reported that both enzyme inducing and non-enzyme inducing AEDs are potentially implicated for the comorbid metabolic bone disease (10, 12).

Bone mineral density (BMD) tests can identify osteopenia/osteoporosis, determine the risk for fractures, and measure the response to osteoporosis treatment. BMD values depend upon bone mineral content (BMC) and bone size. The most widely recognized BMD test is called a dual-energy X-ray absorptiometry (DEXA) (9, 16–18). DEXA is a non-invasive, painless, easy to use, and with less exposure to irradiation. Biochemical indices of bone and mineral metabolism include measurement of blood concentrations of calcium, phosphorus, vitamin D, parathyroid hormone (5, 19–23), and bone remodeling markers [e.g., the receptor activator of nuclear factor-kappa B ligands (RANKL) and osteoprotegerin (OPG)] (24). Such markers may indirectly identify patients with bone disease.

The severity of bone disease with AEDs is heterogeneous. In cases with mild severity, there may be more subtle changes in bone quality and hypovitaminosis D, hypocalcemia, and hyperparathyroidism. Cases of intermediate severity may exhibit characteristic features of osteopenia/osteoporosis. Some patients with severe bone disease may manifest features of osteomalacic disorder (5, 13, 25).

The exact pathophysiological mechanisms of secondary or pathologic bone loss with different AEDs are still uncertain. However, some mechanisms have been suggested, which include hepatic enzyme induction with increase in the metabolism of vitamin D (13–15), impaired calcium absorption (26), direct effect of AEDs on bone cells (9), and resistance to parathyroid hormone and inhibition of calcitonin secretion (27). Identification of exact pathogenic factors involved in development of bone disease with epilepsy and its medications is of primary importance for preventive and treatment strategies.

### Aim of the work

This study was aimed to determine the followings in adult patients with epilepsy and on chronic treatment with AEDs (1) the types and severity of bone disease, (2) the potential mechanism(s) of osteoporosis/osteopenia induced by AEDs as data on this subject are controversial and inconsistent, and (3) the status of OPG/RANKL system (as markers of bone remodeling) and its relationship to BMD and related biochemical parameters of bone disease. The exact role of the OPG/RANKL system and its clinical magnitude in the pathogenesis of bone disease with epilepsy are still uncertain.

## Materials and Methods

This is a comparative cross-sectional case–control study. This study included 75 adults with age ranged between 20 and 50 years and duration of illness of 6–25 years. The patients’ seizure types were diagnosed according to the International League Against Epilepsy (ILAE) criteria (28). Table 1 showed the demographical and clinical features of the studied patients. Patients were recruited from the out-patient epilepsy clinic of the Department of Neurology and Psychiatry of Assiut University Hospital, Assiut, Egypt. All patients were treated with conventional AEDs (CBZ, VPA, or CBZ + VPA) for at least 6 months before participation in this study. Forty healthy subjects recruited from the general population and matched for age- (range: 20–50 years; mean: 30.36 ± 7.59), sex-, and socioeconomic status were also included in this study for statistical comparisons.

**Table 1:** **Demographical and clinical features of the studied patients**.

Age (years)	20–50 (31.90 ± 5.62)
Gender (male/female)	42/33
Body mass index (BMI) (kg/m^2^)	16–40 (25.89 ± 2.69)
Duration of illness (years)	6–25 (12.65 ± 5.42)
Type of epilepsy
GTC	15 (20%)
Complex partial/partial epilepsy with secondary generalization	60 (80%)
AED(s) utilized
CBZ	40 (53.33%)
VPA	23 (30.67%)
Polytherapy (CBZ + VPA)	12 (16%)
Dose of AED(s) utilized (mg/day)
CBZ	400–1200 (650.55 ± 289.83)
VPA	750–1400 (1050.85 ± 560.52)
Duration of treatment (years)	6–20 (10.57 ± 3.55)
Serum drug level (μg/ml)
CBZ	4.5–11.0 (8.02 ± 3.05)
VPA	40.86–105.80 (70.65 ± 35.20)
Degree of control on AED(s)
Controlled (seizure free for ≥1 year)	48 (64%)
Uncontrolled	27 (36%)

*Values are expressed as mean ± SD and number (%)*.

*GTC, generalized tonic-clonic; AEDs, antiepileptic drugs; CBZ, carbamazepine; VPA, valproate*.

Exclusion criteria (for patients and control subjects) included: (1) a history of an overt medical disorder before the onset of epilepsy which likely affect bone metabolism [e.g., thyroid (hyperthyroidism and hypothyroidism), hepatic, renal hematological, rheumatologic and gastrointestinal diseases, hypogonadism, and malabsorption], (2) medications known to affect bone turnover or can contribute to bone loss (e.g., glucorticoids, bisphosphonates, thiazides, anticoagulants, and steroids), (3) intake of calcium or vitamin D supplements, (4) risk factors for osteoporosis [e.g., family history of osteoporotic fracture, estrogen deficiency resulting from early menopause (before age 45 years), either naturally from surgical removal of the ovaries or as a result of prolonged amenorrhea (abnormal absence of menstruation) in younger women, and (5) overt bone deformities (e.g., osteogenesis imperfecta).

Details of the protocol were approved by the local ethics committee and all patients gave written informed consent to their participation.

All patients and healthy control subjects underwent the same research protocol, which included medical, neurological, and endocrinological histories and examinations.

Data collection included age, sex, weight, height, and body mass index (BMI). Seizure variables recorded were: age at onset, duration of illness, type, frequency, type of utilized AED(s) (monotherapy or polytherapy), duration of treatment, degree of patients’ control on AED(s), and obvious side effects from medications. Regarding the degree of control on AEDs, patients were considered controlled on AEDs, when seizure free for ≥1 year.

### Bone mineral density

Bone mineral density was measured using Bone Densitometer, Dual-Energy, DMS Lexxos DR. BMD was expressed as gram per square centimeter and as *T*- and *Z*-scores. *T*-score is the difference in standard deviations between a given bone density value and peak bone density in the normal reference population. *Z*-score is the age- and sex-adjusted *T*-score. When a *T*-score appears as a negative number (such as –1, –2, or –2.5), it indicates low bone mass. The greater the negative number, the greater is the risk of fracture. The World Health Organization (WHO) defines osteoporosis as a *T*-score <−2.5 and osteopenia as a *T*-score between −1.0 and −2.49. Subjects with *T*-scores >−1.0 are considered to have normal BMD (29).

### Laboratory investigations

Subjects included in this study were controlled for timing of blood sampling, which were withdrawn at 8.00–10.00 a.m. after an overnight fast and were seizure free for at least 72 h (as any postictal central neurochemical dysfunction, is recognized to reverse within hours). The samples were centrifuged and serum aliquot and frozen at −70°C till used for different assays. The following laboratory tests were done: (a) *Standardized*, which included: measurements of complete blood count (CBC) and serum concentrations of creatinine, liver enzymes, and fasting blood glucose, and (b) *Specific*, which included measurements of serum calcium, magnesium, phosphate, and alkaline phosphatase (ALP), which were measured using spectrophotometric method (Egypt Company for Biotechnology, Cairo, Egypt). Serum 25-hydroxyvitamin D [25(OH)D] was determined using competitive binding protein assay (Immundiagnostik AG, Blenheim, Germany). Vitamin D deficiency was defined by serum levels of 25OHD <25 nmol/L (10 ng/ml) while insufficiency was defined as a 25OHD between 25 and 50 nmol/L [10–20 ng/ml (30)]. Serum levels of OPG and soluble receptor activator of nuclear factor-kappa B ligand (sRANKL) were measured by enzyme link immunosorbent assay (ELISA) kits, using reagents supplied by Bender MedSystems GmbH (Austria, Europe) and BioVendor Laboratorni medicine (Modrice, Czech Republic). The sensitivities of the kits were 0.125 pmol/L for OPG and 0.2 pmol/L for sRANKL. Concentrations were assessed in combination with the cross-sectional assessment during clinical evaluation and interviewing of patients. The serum levels of AEDs were measured as part of the investigation in batched assays in the Therapeutic Drug Monitoring (TDM) laboratory of Assiut University Hospital, Assiut, Egypt, using Fluorescence Polarization Immunoassay System of Abbott, TDxFLX apparatus (Abbott Lab, Wiesbaden, Germany). The approximated reference therapeutic serum level of CBZ was 4–10 μg/ml and 50–100 μg/ml for VPA (31).

### Statistical analysis

Calculations were done with the statistical package SPSS, version 12.0. Data were presented as mean ± SD or number and percentage. The normal distribution of data was confirmed by calculating skewness and kurtosis before applying standard tests. For comparison of two groups, the non-parametric test for independent variables was used while comparisons of multiple groups were done using ANOVA and Kruskall–Wallis tests for parametric and non-parametric variables, respectively. Chi-square test was used to compare frequency of qualitative variables among different groups. Spearman’s and Pearson’s correlation tests were used for correlating non-parametric and parametric variables. To determine the relationship between BMD and markers of bone turnover, a linear regression analysis was done. For all tests, values of *p* < 0.05 were considered statistically significant.

## Results

This study included 75 adults with primary epilepsy, with mean age of 31.90 ± 5.62 years and duration of illness of 12.65 ± 5.42 years. Patients were treated with one or more conventional AED(s) with nearly three quarter of the patients were on CBZ mono- or polytherapy for 10.57 ± 3.55 years. Nearly, two-thirds of the patients were controlled on the prescribed AEDs. Tables 2 and 3 showed the DEXA results of the studied groups and the frequency of patients with DEXA abnormalities. Compared to control subjects, patients had significantly lower BMD (*p* < 0.001; *p* < 0.0001), BMC (*p* < 0.001; *p* < 0.001), *Z*-score (*p* < 0.0001; *p* < 0.0001), and *T*-score (*p* < 0.0001; *p* < 0.0001) at the femoral neck and lumbar spine, respectively. Abnormalities in more than one site [i.e., femoral neck (unilateral or bilateral) and lumbar spine] were seen in affected patients. The majority of male patients had osteoporosis of the femoral neck and lumbar spine while the majority of females had osteopenia regardless of the type, dose, or serum level of AEDs [versus 20% (*n* = 8) for control subjects with osteopenia in the lumbar spine]. Bone pain (particularly low back pain) was reported in 45 patients (33.75%) particularly in female (*n* = 36, 80%). Twenty patients (15%) had history of limb fracture (males = 13, 65%; females = 7, 35%) before participation in this study. Tables 4 and 5 showed the laboratory results of the studied groups and the frequency of patients with abnormal biochemical parameters. Compared to control subjects, patients had significantly lower serum calcium (*p* < 0.0001), 25(OH)D (*p* < 0.0001), and OPG (*p* < 0.001) and higher ALP (*p* < 0.001), sRANKL (*p* < 0.0001) levels, and sRANKL/OPG (*p* < 0.0001). Nearly, 52% of the patients had hypocalcemia [versus 25% or *n* = 12 control subjects], 93% of the patients had vitamin D deficiency/insufficiency [versus 30% or *n* = 12 control subjects with vitamin D insufficiency], and 31% had high levels of sRANKL and 49% had low levels of OPG. No significant differences were identified between the levels of different laboratory and DEXA results in relation to the age, gender, weight, type, dose, or serum levels of AEDs, mono- versus polytherapy and the degree of control on AEDs. Correlations between demographical-, clinical-, laboratory-, and DEXA parameters showed that in patients with epilepsy (Table 6): (1) duration of illness and BMD at the femoral neck and lumbar spine respectively, (2) the duration of treatment with AEDs were correlated with BMD at the femoral neck and lumbar spine, respectively, levels of calcium, 25(OH)D, sRANKL, and OPG, (3) BMD at the femoral neck and lumbar spine and serum levels of 25(OH)D, sRANKL, and OPG, (4) levels of calcium and 25(OH)D, (5) sRANKL and OPG. In linear regression analysis and after adjusting for gender, age, weight, duration of therapy, and number of AEDs, we observed an association between BMD, 25(OH)D (partial *R*^2^ = 1.5, *p* = 0.04), and sRANKL (partial *R*^2^ = 1.7%, *p* = 0.03) concentrations.

**Table 2:** **Dual-energy X-ray absorption (DEXA) results of the studied groups**.

DEXA results	Patients	Control subjects
**BMD; g/cm^2^**
Femoral neck	0.204–1.132	0.536–0.965
	0.740 ± 0.125**	0.897 ± 0.089
L2–L4	0.483–0.983	0.520–0.983
	0.665 ± 0.209***	0.850 ± 0.137
**BMC; g**
Femoral neck	12.72–52.09	27.99–51.29
	33.76 ± 0.17**	48.28 ± 0.25
L2–L4	16.30–67.39	27.38–57.09
	39.84 ± 0.26**	47.46 ± 0.06
**AREA; cm^2^**
Femoral neck	22.46–76.17	38.77–64.98
	39.96 ± 0.14**	49.75 ± 0.45
L2–L4	30.76–71.27	32.77–67.78
	47.37 ± 0.08**	37.33 ± 0.25
***Z*-SCORE**
Femoral neck	−7.9 to 0.2	−2.2 to 2.0
	−3.9 ± 2.6***	1.5 ± 0.6
L2–L4	−6.6 to 1.9	−1.5 to 2.0
	−4.5 ± 2.1***	1.2 ± 0.3
***T*-SCORE**
Femoral neck	−7.0 to 0.1	−0.6 to 3.0
	−5.6 ± 1.0***	2.44 ± 1.1
L2–L4	−6.6 to −2.0	−0.5–3.0
	−4.43 ± 0.2***	1.5 ± 0.8

*Data are expressed as range and mean ± SD*.

*BMD, bone mineral density; BMC, bone mineral content*.

**Significance: 0.001; ***significance: 0.0001.

**Table 3:** **The frequency of patients with Dual-energy X-ray absorption (DEXA) abnormalities**.

Bone mineral density (BMD)	Femoral neck (*n* = 75)		Lumbar spine (L2–L4) (*n* = 75)
	Unilateral	Bilateral	
Osteopenia (*T*-score −1.0 to −2.49)	18 (24%)	4 (5.33%)	10 (13.33%)
Males	10 (55.56%)	3 (75%)	3 (30%)
Females	8 (44.44%)	1 (25%)	7 (70%)
Osteoporosis (*T*-score <−2.5)	13 (17.33%)	3 (4%)	54 (72%)
Males	9 (69.23%)	3 (100%)	33 (61.11%)
Females	4 (30.77%)	0	21 (38.89%)

**Table 4:** **Laboratory results of the studied groups**.

Laboratory variable	Patients	Controls
Calcium (mg/dL)	5.50–11.00	8.00–10.22
	6.86 ± 1.06***	9.35 ± 1.11
Magnesium (mg/dL)	1.30–5.30	1.60–2.69
	3.54 ± 0.07	4.04 ± 0.52
Phosphorus (mg/dL)	2.21–7.26	2.60–5.56
	4.09 ± 1.01	3.32 ± 0.72
ALP (IU/L)	236.76–965.76	287.04–665.90
	703.54 ± 254.89**	468.75 ± 203.50
25(OH)D (nmol/L)	12.00–55.00	70.00–112.00
	25.67 ± 8.09***	88.70 ±5.94
sRANKL (pmol/L)	4.03–16.49	0.49–5.51
	10.34 ± 3.34***	2.46 ± 1.17
OPG (pmol/L)	0.37–3.73	2.71–4.41
	2.09 ± 0.78**	3.96 ± 0.59
sRANKL/OPG ratio	1.08–37.22	0.15–2.03
	6.55 ± 0.13***	0.73 ± 0.43

*Data are expressed as range and mean ± SD*.

*ALP, alkaline phosphatase; 25(OH)D, 25-hydroxyvitamin D; sRANKL, soluble receptor activator of nuclear factor-kappa B ligand; OPG, osteoprotegerin*.

**Significance: 0.001; ***significance: 0.0001.

**Table 5:** **The frequency of patients with abnormal biochemical parameters**.

Laboratory variable	Patients (*n* = 75)
Calcium	39 (52%)
Magnesium	8 (10.67%)
Phosphorus	4 (5.33%)
ALP	32 (42.67%)
25(OH)D
Vitamin D deficiency (<25 nmol/L)	50 (66.67%)
Vitamin D insufficiency (25–50 nmol/L)	20 (26.67%)
sRANKL	23 (30.67%)
OPG	29 (38.67%)

*ALP, alkaline phosphatase; 25(OH)D, 25-hydroxyvitamin D; sRANKL, soluble receptor activator of nuclear factor-kappa B ligand; OPG, osteoprotegerin*.

**Table 6:** **Correlations between demographic-, clinical-, laboratory- and DEXA parameters**.

Variables	Duration of illness	Duration of treatment	Calcium	25(OH)D	sRANKL	OPG
BMD						
Femoral neck	−0.360	−0.505	–	0.476	−0.478	0.480
	**0.043**	**<0.001**	–	**0.042**	**0.005**	**0.006**
L2–l4	−0.432	−0.301	–	0.360	−0.360	0.330
	**0.010**	**0.012**	–	**0.010**	**0.010**	**0.010**
Calcium	–	−0.455	–	0.386	–	–
	–	**0.005**	–	**0.005**	–	–
25(OH)D	–	−0.655	0.386	–	–	–
	–	**<0.001**	**0.005**	–	–	–
sRANKL	–	0.387	–	–	–	−0.725
	–	**0.007**	–	–	–	**<0.001**
OPG	–	−0.525	–	–	−0.725	–
	–	**<0.001**	–	–	**<0.001**	–

*BMD, bone mineral density; 25(OH)D, 25-hydroxyvitamin D; sRANKL, soluble receptor activator of nuclear factor-kappa B ligand; OPG, osteoprotegerin*.

## Discussion

Although the knowledge regarding disease- and AEDs-related bone abnormalities are rapidly increasing; however, data regarding the mechanisms of bone loss in patients with epilepsy are not clearly understood. In this study, the potential relationships between the BMD findings in a group of ambulatory adults with epilepsy and treated with AEDs and markers of bone remodeling, which include calcium, 25(OH)D, ALP, and OPG/RANKL systems were discussed. The results of this study showed that in ambulatory adults with epilepsy and on chronic treatment with AEDs, there is an increased risk of (1) bone loss (osteopenia/osteoporosis) and fracture, (2) hypocalcemia (52%) and 25(OH)D deficiency/insufficiency, (3) increased bone turnover as indicated by lower serum levels of OPG and higher levels of sRANKL and sRANKL/OPG ratio (markers of bone remodeling), and (4) metabolic bone compromise with prolongation of the duration of treatment with AEDs due to disturbed mineral metabolism associated with compromise of bone remodeling with accelerated bone turnover. These changes occur regardless of patients’ age, gender, weight, type, dose, or serum levels of AEDs, number of utilized AEDs, and the degree of control on AEDs, but BMD loss was also associated with prolongation of the disease duration which also indicated prolonged duration of treatment.

First, the findings of this study showed increased markers of bone loss as evidenced by lower BMD, BMC, *Z*-score, and *T*-score at the femoral neck and lumbar spine regardless of the type, dose, or serum level of the utilized AED. Osteoporosis of the femoral neck was reported in 21.33% while that of the lumbar spine was reported in 72%. Osteopenia of the femoral neck was reported in 29.33% while that of the lumbar spine was reported in 13.33%. No significant differences in DEXA results were identified between patients on CBZ, VPA, or polytherapy. Bone mass changes reported in the literature vary greatly for patients undergoing treatment with AEDs and ranged between 13 and 75%. However, both enzyme inducing and non-enzyme inducing AEDs are potentially implicated for bone mass loss with epilepsy (10, 12–15, 20). The majority of male patients had osteoporosis of the femoral neck and lumbar spine while the majority of females had osteopenia. The variations between the results of different studies may depend on the type of AEDs used, and the AED blood level required for effective treatment. Other factors may include the length of time of treatment, the existence of associated pathologies, the age and sex of the patients, their level of physical activity, and where and how the measurements are made. We reported history of fracture in 15%, which was predominant in males (7, 32). It has been reported that gender plays a major role in the pathogenesis of fractures. In accordance, Cummings et al. (33) reported that fractures commonly occurred in males aged <40 years (between 30 and 49) and then rapidly declined while the peak rate occurred 20 years later (between age 50 and 59 years) in females, and declined subsequently.

Second, the findings of this study showed that biochemical abnormalities of bone-related mineral metabolism included hypocalcemia (52%) and hypovitaminosis D (93.33%). 25(OH)D levels were positively correlated with calcium levels but negatively correlated with BMD. 25(OH)D deficiency/insufficiency was reported irrespective to the type of AED (17). Hypovitaminosis D was previously reported in 50% of ambulatory patients on AEDs. The other common laboratory abnormalities described in relation to AED are elevated levels of ALP and hyperparathyroidiam (5, 13, 17, 19, 20, 34). It has been suggested that hepatic induction of cytochrome P450 enzymes by enzyme inducing AEDs (e.g., phenobarbital, phenytoin, and CBZ) increases the metabolism and clearance of vitamin D with secondary hypocalcemia and hyperparathyroidism (13–15). The mechanism of disturbed mineral metabolism due to VPA is not completely understood. VPA may cause renal tubular dysfunction, which could indirectly influence bone and mineral metabolism with increased urinary loss of calcium and phosphorus. In support, VPA has been associated with reversible Fanconi syndrome (35). It is known that approximately 99% of calcium in the body is stored in the skeleton. Calcium is stored in or removed from the skeleton to regulate serum calcium levels. 25(OH)D is the major circulating form of vitamin D. 25[OH]D is biologically inert and transported to the kidney, where renal 1a-hydroxylase converts 25(OH)D to 1,25(OH)2D. 1,25(OH)2D is an important regulator of serum calcium concentration. 1,25(OH)2D directly enhances the entry of calcium through the plasma membrane into intestinal absorption cells and transfers calcium across the basolateral membrane into the circulation to maintain calcium homeostasis. 1,25(OH)2D promotes mineralization of osteoid and, with appropriate levels of calcium and phosphorus, induces deposition of calcium hydroxyapatite into the bone matrix (36). Controversial results reported lack of any correlation between vitamin D and BMD in patients with epilepsy in some studies (4, 37). Some suggested that AEDs may inhibit vitamin D-mediated calcium absorption (26). Filardi et al. (38) reported that adults who were on chronic anticonvulsant therapy and who lived in low latitude regions had normal bone mineral density as well as vitamin D serum level. Mattson and Gidal (14) reported low BMD irrespective of vitamin D levels. Verrotti et al. (22) reported that VPA doses do not seem to change bone turnover in adult epileptic patients. This could be explained by vitamin D-independent pathways such as impaired calcium absorption (26), resistance to parathyroid hormone and inhibition of calcitonin secretion (27), direct effects of AEDs on bone cells (9), hyperparathyroidism and calcitonin deficiency (39).

Third, this study showed reduced levels of OPG and higher levels of sRANKL and sRANKL/OPG ratio in patients with epilepsy, the duration of treatment with AEDs were positively correlated with sRANKs and negatively correlated with OPG, BMD at the femoral neck, and lumbar spine and serum levels of sRANKL and OPG and in linear regression analysis and after adjusting for gender, age, weight, duration of therapy, and number of AEDs, we observed an association between BMD, 25(OH)D, and sRANKL concentrations.

Bone is a complex dynamic tissue. It undergoes remodeling, a continuing process of bone resorption and formation in discrete areas throughout the skeleton. BMD represents a coordinated action or the balance between bone resorption or the bone-depleting action of osteoclasts and the bone-formative action of osteoblasts (bone remodeling). Osteoclast and osteoblast (main bone cells) dynamics, body weight, exercise, vitamin D, calcium, and phosphorous homeostasis as well as connective tissue arrangements can alter bone structure and architecture. Several hormones, polypeptides, cytokines, growth factors, nutrients, minerals, and essential cofactors, regulate bone remodeling. The active metabolite of vitamin D [1,25(OH)2D] and adequate amounts of calcium and phosphorus are required for bone formation (40). Bone remodeling is intimately related to changes in expression of members of tumor necrosis factor (TNF) receptor superfamily on bone marrow stromal cells [e.g., the macrophage colony-stimulating factor (M-CSF); the receptor activator of nuclear factor-kappa B ligands (RANKL), RANK and OPG] and various cytokines and calciotropic hormones (41). RANKL binds the RANK receptor on osteoclast precursors and induces the formation of osteoclasts by a special signaling pathway (42, 43). A soluble form of RANKL (sRANKL) is released from its membrane bound state by metalloproteinases. OPG blocks the RANKL–RANK interaction by binding to and neutralizing both soluble and cell-bound forms of RANKL thus inhibits osteoclast differentiation and activation (44). It is the balance between the expression of RANKL and OPG that determines the extent of bone resorption. It has been suggested that in patients with epilepsy and in order to improve calcium absorption when the 25-OH Vitamin D level is low, 1,25OHD is increased, a conversion step mediated by parathormone. Elevation of the active metabolite of vitamin D, 1,25OHD, can stimulate bone resorption through the receptor-activated NF-KB ligand (RANKL), promoting maturation of osteoclasts (24). In support, bone biopsies from patients with epilepsy revealed diminished volume of trabecular bone and increased amount of osteoclastic resorptive activity and osteoid in 10 and 40% of patients receiving long-term AED therapy (37).

Despite the strength of this study, it has some limitations: (1) the recruitment of the study group from a tertiary care center with more severe cases and this explains the high percentage of observed bone loss evidence by DEXA and the biochemically related abnormalities, (2) because of the cross-sectional nature of the present study, it was not possible to know temporal relationship between bone loss to the use of AEDs. However, such limitations could only be overcome through a longitudinal and multi-center study design.

## Conclusion

Ambulatory adults with epilepsy and chronic treatment with AEDs are at greater risk of metabolic osteopathy as evidenced by osteopenia/osteoporosis, fracture, hypocalcemia, and hypovitaminosis D. The metabolic bone loss and mineral abnormalities are associated with accelerated bone turnover as evidenced by higher levels of sRANKL and sRANKL/OPG ratio and lower levels of OPG (markers of bone remodeling). Thus, patients receiving long-term AED have to be monitored for indices of bone health, including BMD and vitamin D status and to be provided with proper preventive and treatment strategies.

## Conflict of Interest Statement

The authors declare that the research was conducted in the absence of any commercial or financial relationships that could be construed as a potential conflict of interest.

## Abbreviations

25(OH)D, 25-hydroxyvitamin D; AEDs, antiepileptic drugs; ALP, alkaline phosphatase; BMC, bone mineral content; BMD, bone mineral density; CBZ, carbamazepine; OPG, osteoprotegerin; sRANKL, soluble receptor activator of nuclear factor-kappa B ligand; VPA, valproic acid.
